# Looking into the flora of Dutch Brazil: botanical identifications of seventeenth century plant illustrations in the *Libri Picturati*

**DOI:** 10.1038/s41598-021-99226-8

**Published:** 2021-10-05

**Authors:** Mireia Alcàntara-Rodríguez, Mariana Françozo, Tinde Van Andel

**Affiliations:** 1 Faculty of Archaeology, Leiden University, Einsteinweg 2, 2333 CC Leiden, The Netherlands; 2 Faculty of Archaeology, Associate Professor in Museum Studies, PI ERC BRASILIAE Project, Leiden University, Leiden, The Netherlands; 3 Institute for Biology, Clusius Chair in History of Botany and Gardens, Leiden University, Sylviusweg 72, 2333 BE Leiden, The Netherlands; 4 Naturalis Biodiversity Center, PO Box 9517, 2300 RA Leiden, The Netherlands

**Keywords:** Ecology, Plant sciences

## Abstract

The *Libri Picturati* includes a collection of plant illustrations from seventeenth century Dutch Brazil that is kept in the Jagiellonian library in Krakow since World War II. While many studies focused on the artistic details and history of these images, we identified the flora depicted. We used contemporary textual sources (e.g., *Historia Naturalis Brasiliae*), monographs and taxonomist’ assessments. We checked origin, life form, domestication and conservation status and the plant parts that are represented. We identified 198 taxa, consisting mostly of wild, native rainforest trees and 35 introduced species. Fertile branches are the most represented, although some loose dry fruits and sterile material were also painted, which sheds light into the collection methods by naturalists in Dutch Brazil. Several species are no longer abundant or have become invasive due to anthropogenic influences since colonialism. Through this botanical iconography, we traced the first records of the sunflower and the Ethiopian pepper in Brazil, as well as the dispersion and assimilation of the flora encountered in the colony by Indigenous, African and European peoples. We emphasized the relevance of combining visual and textual sources when studying natural history collections and we highlighted how digitalization makes these artistic and scientific collections more accessible.

## Introduction

“We are also told that the nature of the foreign was understood through its natural objects. What this nature was, we are often left to wonder”^1^.

In 1977, in the Jagiellonian library of Krakow (Poland), zoologist Peter Whitehead found a treasure that most scholars had already considered lost: a collection of Brazilian illustrations in the *Libri Picturati*^2,3^. The *Libri Picturati* consists of thousands of drawings and paintings of flora, fauna and people from several ethnical backgrounds, which were bound together in the nineteenth century^4^. It contains—inter alia—sixteenth century plant watercolors, attributed to the circle of correspondents around Carolus Clusius and Charles de Saint Omer^5^ and a collection of Brazilian Natural History illustrations made during the Dutch occupation of northeast Brazil. The latter were created under the patronage of count Johan Maurits of Nassau-Siegen (governor-general of Dutch Brazil from 1636 to 1644) by artists and naturalists who aimed to represent the natural elements that surrounded them in the colony. The Brazilian collection includes the seventeenth century oil paintings known as the *Theatrum Rerum Naturalium Brasiliae* (from now on *Theatrum*), the watercolor drawings known as Handbooks, *Manuais* or as the *Libri Principis* (*LP*) and the crayon/pencil sketches and oil paintings bound in the *Miscellanea Cleyeri* (*MC*)^4^.

Other contemporary materials to these visual sources of Brazilian nature include an account of Johan Maurits’ endeavors in Brazil written by polymath Caspar Barlaeus^6^; the encyclopedia *Historia Naturalis Brasiliae* (from now on HNB) authored by naturalist Marcgrave and physician Piso and published by Johannes de Laet in 1648^7^; and the *India Utriesque re Naturali et Medica* (IURNM), a modified and doubtful version of the HNB^8^. Both the HNB and IURNM contained descriptions and woodcut images of animals, plants, people and tropical diseases of Dutch Brazil. These textual sources were published in the Netherlands and circulated widely among European naturalists and other inquiring minds interested in the ‘exotic’ nature of the Americas, while the Brazilian images were exchanged as diplomatic gifts^3^. In 1652, Johan Maurits sent these images to Frederick William, Elector of Brandenburg (1620–1680), who passed them to his court physician Christian Mentzel^4^. Also interested in the natural world, Mentzel devoted himself to assemble all the illustrations from 1660 to 1664. This resulted in a fairly organized art collection of Brazilian nature that was kept in the Elector’s library (presently part of the *Staatsbibliothek zu Berlin*) available for study to humanists and intellectuals alike. Almost 300 years later, this collection was evacuated during World War II to protect it from the bombings. The *Libri Picturati* went through several transfers and ultimately, the Brazilian iconography was found in Poland, which opened new possibilities for research^3,4^.

### Previous research and our role

When the paintings were still in Berlin, Lichtenstein^9,10^ studied the fish and other animals depicted, while Schneider^11^ identified the birds and matched them with the HNB woodcuts. Martius^12^ used the *Theatrum* to compare its plant images to the HNB and IURNM, elaborated further on Brazilian botany in his commentaries on Marcgrave’s plants, and described this as “such a laborious task”^13^^: XI^. Albertin^4^ focused on the content of the *Theatrum* from an art historical perspective with special focus on the authorship of the images. Whitehead and Boeseman^3^ provided a meticulous overview of the visual and textual sources produced in or about Dutch Brazil, and considered the *Theatrum* images to be the basis for the HNB woodcuts. Brienen^14,15^ focused on the relationship between visual information and scientific enterprise in the early modern period and advanced hypothesis about the authorship of the images. Ferrão and Soares^16,17^ historically contextualized and reproduced the images of the *Libri Picturati*, while commenting on some of the illustrations. Recently, Scharf^18^ analyzed the different pictorial techniques behind the composition of the *LP*. The illustrations were clearly made by different artists using various techniques, but the authorship is still uncertain. Scholars have attributed them to Dutch painters Albert Ekchout^14,19,20^ or Frans Post^11^. Others suggested that they were made by Marcgrave, who had taken artistic training and produced several drawings that served as the basis for some of the woodcuts depicted in the HNB^3^. Scharf^18^ suggested count Johan Maurits as the artistic hand behind some of the watercolors. Hence, these illustrations have been mostly studied from an (art) historic approach. We chose to identify the species depicted in the paintings, thereby revealing their domestication status, geographic origin and the natural vegetation type in which they occur, to suggest where they were made and how close naturalists and artists collaborated in Dutch Brazil.

A team of art historians and botanists that studied the sixteenth century illustrations of the *Libri Picturati* have identified the plants depicted^21^, but this has not been the case for the Brazilian collection. In the *Theatrum*, there are some taxonomic notes written above the plant illustrations—of dubious authorship, which Albertin^4^ attributed to Lichtenstein. Nevertheless, these are often incomplete, outdated or erroneous. The recent digitization of all plant illustrations by the Jagiellonian library has greatly facilitated research on these valuable historic images. Here we present the first botanical revision and systematic identifications of the plants depicted in the Brazilian collection of the *Libri Picturati*: the *Theatrum Rerum Naturalium Brasiliae*, the *Libri Principis* and the *Miscellanea Cleyeri*. We posed the following research questions: (1) What plant taxa are illustrated in the *Libri Picturati*? (2) What taxa were intended to be added to the original collection by Mentzel, but lack illustrations in this collection? (3) What insights can we infer from the illustrations on the methods of plant collection and collaboration between naturalists and artists in the colony? (4) What are the differences and similarities in botanical content between visual and written sources? Brienen^14^ and Ferrão and Soares^16^ attested that the flora represented in the *Libri Picturati* came mostly from the surroundings of Recife, the capital of the colony. Due to Marcgrave’s multiple expeditions to the interior of Brazil^22^, we expected to find plant species from a wide variety of ecosystems in northeastern Brazil: the semi-arid *sertão* or *caatinga* and the Atlantic rainforest, which includes savannas, mangroves, and dry shrub land^23^^: 1070^. Following Whitehead and Boeseman^3^ and Teixeira^19^, we likewise expected to find similar plant taxa in the *Libri Picturati* as in Marcgrave and Piso’s natural history treatises, because both visual and textual representations were made in the same area around the same time in Dutch Brazil.

## Methods

Due to Covid-19 travel restrictions, we were not able to study the original material in Poland. Jagiellonian library curator Izabela Korczyńska provided the scanned images of the *Theatrum*, while the *LP* (https://jbc.bj.uj.edu.pl/dlibra/publication/193892/edition/183824/content) and *MC* (https://jbc.bj.uj.edu.pl/dlibra/doccontent?id=197455) were retrieved from Jagiellonian Digital Library. We systematized all information on the botanical images in a spreadsheet organized by page number, vernacular plant names and reference annotations to the HNB and IURNM on the folios. Next, we included our taxonomical identifications (to species level whenever possible), and indicated whether these taxa were present in Marcgrave and Piso’s books^7,8^ or Marcgrave’s herbarium (1638-1643/4) (available at https://samlinger.snm.ku.dk/en/dry-and-wet-collections/botany/general-herbarium/the-marcgrave-herbarium/). We identified the illustrated plant taxa by using our previous identifications of the species present in the HNB, the IURNM and Marcgrave’s herbarium^24^, and previous research of the Brazilian botanists Pickel^25^ on the species described in Marcgrave and Piso’s textual sources and Andrade-Lima et al.^26^ on Marcgrave’s herbarium. We compared the illustrations with related seventeenth century Brazilian images (Wagener, c.1641^27^). In addition, we used literature on the Brazilian flora^28,29^, monographs^30–32^, online herbarium databases, such as the Global Biodiversity Information Facility (https://gbif.org/), Tropicos (https://tropicos.org/), Naturalis Biodiversity Center (https://bioportal.naturalis.nl/). We also consulted expert taxonomists in Brazil, the Netherlands, Mexico, and the United States.

Information on the native or introduced status and life form of the depicted taxa was retrieved from the online Flora do Brazil 2020 (http://floradobrasil.jbrj.gov.br/). We checked the origin of the exotic species by using the database Pl@ntUse (https://uses.plantnet-project.org/en/Main_Page). We also checked other sources on the introduction of specific taxa to Brazil (e.g.,^33–36^). We noted the plant parts represented in the illustration, as these give an idea of the collection methods in the mid-seventeenth century. We verified the domestication status of each depicted taxon from Marcgrave and Piso’s books^7,8^, archaeobotanical and historical studies on pre-Columbian plant domesticates^37–39^ and research on Johan Maurits’ palace gardens in Recife^40^ based on the account by Barlaeus^6^. Although the varying degrees in the level of domestication of certain species fit better in a continuum^37^, for the purposes of this paper we distinguish three categories: wild, cultivated and domesticated. Following Levis et al.^38^^: 6^, we considered domesticated species those that *show substantial morphological and genetic changes and depend on human management for their long-term survival*. We included as cultivated species those that are managed to some extent by humans, albeit they can also survive and reproduce without them in the wild^38^^: 6^. The conservation status and endemic status were retrieved from the Brazilian database on Flora Conservation (CNC-Flora: https://cncflora.jbrj.gov.br/portal/) and the IUCN Red List (https://iucnredlist.org/). We also referred to the CITES list to check which species are currently protected to avoid over-exploitation by international trade (https://cites.org/). To reconstruct the floristic content of the *Theatrum* if Mentzel had been able to add all plant illustrations he aimed, we verified reference annotations (vernacular names and page numbers) on the empty folios to Marcgrave and Piso’s books^7,8^ and identified the taxa that were meant to be represented on those folios. To update the taxonomical nomenclature, we used the Flora do Brazil 2020 (https://floradobrasil.jbrj.gov.br/) and the Plants of the World Online (https://plantsoftheworldonline.org/).

## Results

### Botanical content of the *Libri Picturati* Brazilian collection

Our identifications of all plant illustrations are listed with their vernacular names, page numbers, and associated information on growth form, geographical origin, conservation and domestication status in Supplementary Dataset S1. From the entire collection of Brazilian plant illustrations in the *Libri Picturati*, we identified 198 taxa that are organized in the *Theatrum*, *LP* and *MC* as indicated in Supplementary Table S1. Between folios 729 and 731 of the *Theatrum*, an illustration of a tea plant (*Camellia sinensis* (L.) Kuntze) is glued, which was sent by Cleyer from Batavia (currently Jakarta, Indonesia), the headquarters of the Dutch East Indian Company. As it was inserted later in the *Theatrum* and not depicted in Brazil, we did not include it in our analysis. A few plants remained unidentified due to a lack of morphological characters, the limited quality of the drawing and/or the lack of references to written sources by Marcgrave or Piso^7,8^.

Among the *LP* botanical watercolors, we identified 34 vascular plant species (38 images) with the Passifloraceae as the most represented family (five species, six images), followed by the Fabaceae (five species, five images). Among the *MC* plant drawings, we identified 26 vascular plant species (34 images) and the most represented families were the Cucurbitaceae (three species, seven images) and the Myrtaceae (three species, three images). Among the illustrated content of the *Theatrum*, we identified 162 vascular plant species (175 images) and one basidiomycete fungus (*Copelandia cyanescens* (Sacc.) Singer, Bolbitiaceae). Fungi were commonly placed within the plant kingdom until the mid-twentieth century. The most represented families among the illustrated content are the Fabaceae (22 species, 22 images), followed by the Solanaceae (10 species, 11 images), Lamiaceae (six species, six images) and Myrtaceae (six species, eight images). The Fabaceae is the most diverse plant family in the world^41^, while the Myrtaceae is one of the most rich-species woody plant family in the Atlantic Forest in Brazil^42^.

### Mentzel’s unfinished task: the intended botanical content of the *Theatrum*

**Figure 1:**
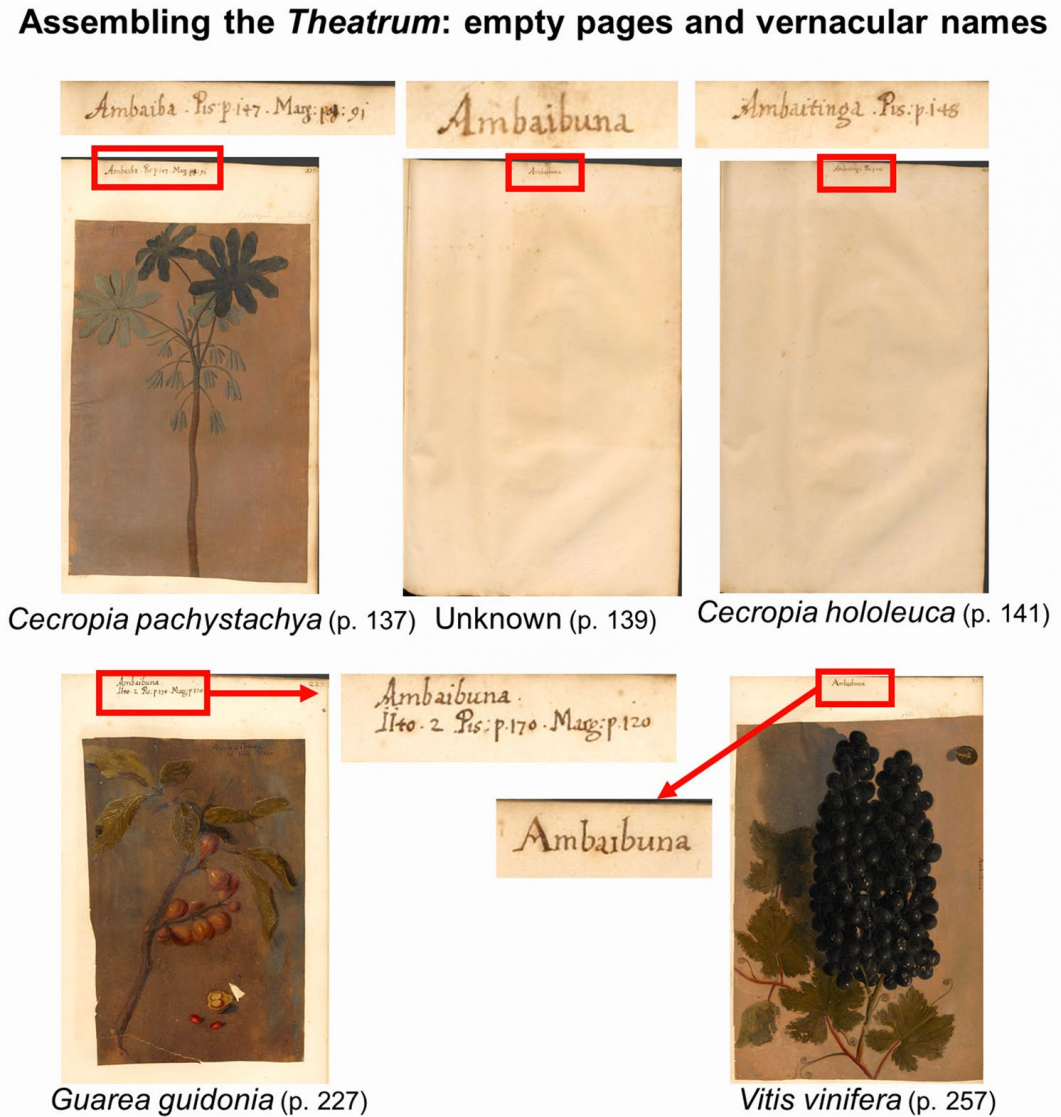
Similar vernacular names for related taxa and distinct taxa associated to the same vernacular name in the *Theatrum Rerum Naturalium*.

The *Theatrum* also includes 206 empty folios, interleaved between 160 folios with plant illustrations (see example in Fig. 1). On most folios, vernacular names and references to the pages of the HNB and IURNM are written on the top center, often relating to one taxon, but sometimes referring to two taxa (Fig. 1). This occurs specially at the end of the collection, as if the maker had ended up with little space and somehow had to squeeze them in. Among these unillustrated folios, the vernacular plant names and references to Marcgrave and Piso’s sources allowed us to identify 196 vascular plant species (218 records) including five ferns from the families Drypteriaceae (one species), Polypodiaceae (one species) and Pteridaceae (three species); one alga (*Sargassum tenuissimum* (Endlicher & Diesling) Grunow, Phaeophyceae) and a marine sponge (*Clathria* cf. *nicoleae* Vieira de Barros, Santos & Pinheiro, Microcioniadae) (Supplementary Dataset S1). Considering that the study of spongiology (Porifera) did not develop until the mid-nineteenth century, these animal colonies must have been considered an aquatic plant because of the tree-like shape and the fact of living attached to the seabed. The most represented family that would correspond to the empty folios was the Fabaceae (29 unillustrated species, 33 records), followed by the Arecaceae (nine species, ten records), Solanaceae (nine species, nine records), and Asteraceae (seven species, seven records). Estimates of the intended botanical content (i.e., empty folios with references together with the illustrated folios) are shown in Table 1.

**Table 1:** Estimations of the botanical content of the *Theatrum Rerum Naturalium*, including empty and illustrated folios.

*Theatrum*	Unfinished plant content	Illustrated plant content	Total (intended) botanical content*
Folios	206	160	366
Illustrations	0	172	172
Vernaculars/plants	220	176	396
Taxa	205	163	335
Species	197	150	313
Genera	5	8	13
Unidentified taxa	3	5	8
Families	74	68	95

*The sums of taxa, species and families exclude the repeated records in both empty and illustrated folios.

On p. 139 of the *Theatrum*, the vernacular name *Ambaibuna* is written on an empty page without reference to Marcgrave’s or Piso’s books. The page with *Ambaibuna* is located between *Ambaiba* (p. 137), which corresponds to the illustration of *Cecropia pachystachya* Trécul, and a blank page with only the vernacular name *Ambaitinga* (p. 141), which corresponds to *C. hololeuca* Miq.^7^^: 92,^^24^ (Fig. 1). The Brazilian *Cecropia* species are known in Tupi-related languages as *Ambauba*, *Ambauva* or *Umbaúba* (https://dataplamt.org.br/), which are phonetically and morphologically similar to *Ambaibuna*. For those reasons, we initially assumed that *Ambaibuna* referred to a *Cecropia* species, but the same name *Ambaibuna* is later repeated together with the name *Iito* (p. 227) next to an illustration that represents a completely different tree species: *Guarea guidonia* (L.) Sleumer (Fig. 1). Furthermore, the name *Ambaibuna* is also written above the illustration of a grapevine, *Vitis vinifera* L. (p. 257), also unrelated to *Cecropia* (Fig. 1).

Whether *Ambaibuna* was a generic name to designate several non-related species or represents a mistake by the author who wrote the names on the illustrations remains unknown. On the other hand, neither Marcgrave nor Piso mentioned *Ambaibuna* in their descriptions of the Brazilian flora. Aside from Marcgrave and Piso’s books^7,8^, it is yet to be determined which source(s) Mentzel relied on when arranging the botanical content of the *Theatrum*. It is nonetheless clear that he must have been confused by the similarity of some of the Tupi-related plant names. Unfortunately, Marcgrave was no longer present to help him match the illustrations, names and descriptions, because he died about 16 years before Mentzel started organizing the Brazilian plant illustrations.

### Origin of the exotic species in the *Libri Picturati*

**Figure 2:**
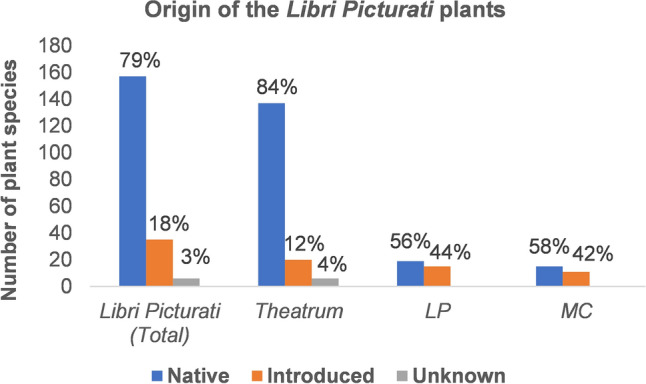
Proportion of native and introduced species in the Brazilian collection of the *Libri Picturati*: *Theatrum Rerum Naturalium* (*Theatrum*), *Libri Principis* (*LP*) and *Miscellanea Cleyeri* (*MC*).

The *Libri Picturati* collection depicts in its majority native Brazilian plants. Most of the species represented in the *Theatrum* are native from Brazil, but the proportion of native species is much lower in the *MC* and lowest in the *LP*, in which almost half of the illustrations represent introduced species (Fig. 2)*.*

There are 35 species of exotic origin in the complete Brazilian collection of the *Libri Picturati* (Supplementary Table S2). These introduced species now occur in (sub-) tropical areas around the world. Most of the exotic plants originally came from other parts of the Americas, especially Mexico, the Caribbean and the Andes region (14 species); followed by those that originated in the African continent (10 species) and tropical Asia (nine species) (Supplementary Table S2). Most of the exotic American plants that were introduced to Brazil were domesticated and traded by indigenous groups long before the European colonization, such as papaya (*Carica papaya* L.), cotton (*Gossypium barbadense* L.), sweet potato (*Ipomoea batatas* (L.) Lam*.*), beans (*Phaseolus vulgaris* L.)*,* guava (*Psidium guajava* L.) and maize (*Zea mays* L.)^37^. Most of the species of Asiatic origin were already naturalized or cultivated in Africa and introduced to Brazil by means of the Trans-Atlantic slave trade before the Dutch arrived, such as yams (*Dioscorea alata* L.), plantains (*Musa* × *paradisiaca* L.) and weeds like *Abrus precatorius* L. and *Plumbago zeylanica* L.^43,44^. Others were introduced from Europe by merchants and settlers, such as the Portuguese Jesuits, who incorporated them as remedies into their *boticas* (Jesuit pharmacies in the colonies). For example, the various *Citrus* and pomegranate fruits were not only planted as fruits but also used to expel roundworms and to combat cold fevers, respectively^45^^: 88^. Before their arrival to Brazil, the Portuguese and Dutch must have been familiar with some African plants, such as *Aloe vera* (L.) Burm.f*.*, *Ricinus communis* L. and *Tamarindus indica* L. These useful plants were already known in Europe through Arabic and Greek medical texts, which knowledge was boosted by their translations into Latin during the High Renaissance^45,46^. *Punica granatum* L. was introduced into the Iberian Peninsula via ancient merchant routes in the Mediterranean^47^ and brough to Brazil by the Portuguese^45^. Grapes (*Vitis vinifera* L.) were already cultivated by the Portuguese in Pernambuco around 1542^48^. Along the Atlantic coast, lemons, pomegranates and grape vines adapted to the new environmental conditions and thrived in the vicinities of Johan Maurits’ residence, as evidenced by the illustrations in the *Theatrum* and textual accounts^6,7,40^.

**Figure 3:**
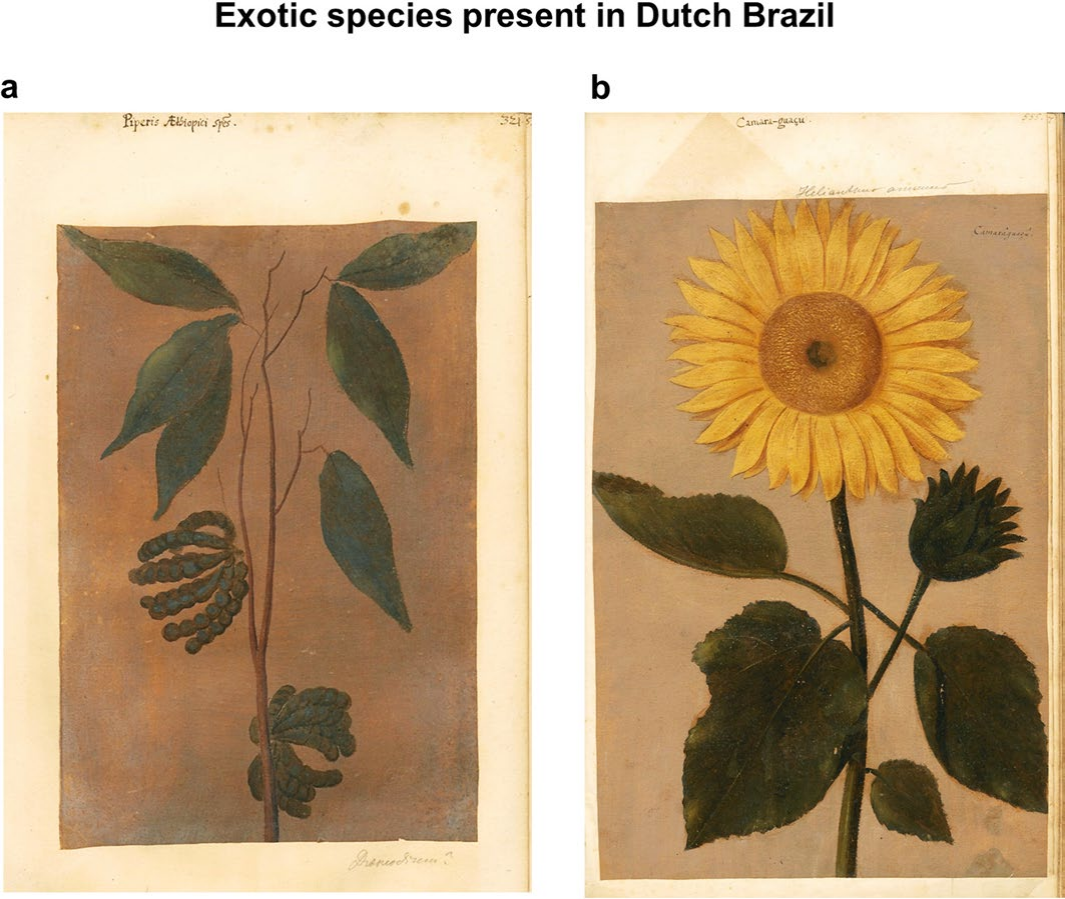
(**a**) The African spice-producing tree *Xylopia aethiopica* depicted in the *Theatrum Rerum Naturalium* (p. 321); (**b**) The first record of the sunflower (*Helianthus annuus*) in Brazil (*Theatrum*: 555).

The presence of these globally commodified plants is common today in Brazil as in many regions worldwide. Other species seem to have lost their popularity over time. The so-called Ethiopian, Guinean or Negro pepper, *Xylopia aethiopica* (Dunal) A.Rich., was present around the 1640s in northeast Brazil, as evidenced in the *Libri Picturati* by a painting with a fruiting branch with leaves named *Piperis aethiopici spés* (Fig. 3a). The first iconography of this aromatic tree in Europe is found in Matthioli’s commentaries on Dioscorides under the name of *Piper aethiopicum*^49^^: 575^ and its fruits were previously cited by the Persian polymath Avicenna (980–1037)^30^. In Europe, this African pepper was commonly used until southeast Asian spices gained popularity in the sixteenth century^50^. In the plantation societies of tropical America, *X. aethiopica* constituted a food crop for enslaved Africans in the early colonial period^43^^: 135^. Today, its fruits are used in aphrodisiac tonics^51^ and special dishes prepared for African deities (Orishas) in Cuba^43^^: 90^, but it is unclear whether the species grows in Brazil. Its current distribution range encompasses West, Central and Southern Africa (https://gbif.org/occurrence/map?taxon_key=3157151). The dry fruits are used in tropical Africa as a condiment, in rituals and as medicine to treat cough, bronchitis, rheumatism, malaria, amenorrhea and uterine fibroids^52–54^. There is an herbarium record in Brazil made by photographer and anthropologist Pierre Verger. The label on the specimen mentions ‘Brazil’ and ‘Plantas de Candomblé’ and it indicates that the voucher was deposited at the Herbarium Alexandre Real Costa (ALCB, according to *Index Herbariorum*: http://sweetgum.nybg.org/science/ih/, accessed 23 August 2021) in Bahia (Verger s/n, ALCB012478, available at ALCB, via Species Link: https://specieslink.net/search/, accessed 23 August 2021) Verger presumably collected this specimen in Bahia in 1967 while he was researching on ritual and medicinal plants used in Candomblé (http://inct.florabrasil.net/alcb-resgate/, accessed 2 June 2021)^55^. However, it seems to be a mixed collection, as the leaves are oppositely arranged and with long petioles, which is uncommon to Annonaceae^30^. In Brazil, the fruits of the Brazilian relative *Xylopia aromatica* (Lam.) Mart. have probably served as a good substitute for *X. aethiopica*, as they have a similar peppery taste and stomachic properties^56^^: 3^, and are more easily gathered from the *cerrado* savannahs or the Amazon rainforest. Voeks^57^ documented *X. aethiopica* seed powder as used in Candomblé rituals by Yoruba practitioners in Bahia. Nevertheless, there is no clear information whether *X. aethiopica* is cultivated in the Neotropics or imported; thus, the origin of the fruits, seeds or its powder in Brazil remains uncertain.

The first reference to the sunflower (*Helianthus annuus* L.) in Brazil dates to the twentieth century, when it was introduced by European immigrants due to its economic value as an oil-producing crop^58^. Sunflowers are of North American and/or Mexican origin ^59,60^, and were introduced to Europe in the sixteenth century by the Spanish, as part of the Columbian exchange^61^. Merchants observed how native Americans benefited from this plant and exported the sunflower to Europe, where it was primarily valued as ornamental and later as a food crop, propelled by genetic improvement by the Russians in the 1800s^59^. Before the sunflower became a popular and well-stablished crop in the twentieth century, this plant was already encountered in northeast Brazil, as evidenced by the illustration in the *Theatrum* (Fig. 3b). Portuguese sailors may have played a role in its introduction to Brazilian territories or it could have been intentionally brought by merchants or Jesuits, although the latter paid more attention to medicinal plants^45,62^. We may also consider the Dutch as active agents in its introduction to their colonies in the northeast. A relevant female agent in the dissemination of the sunflower in the Netherlands was Christine Bertolf (1525–1590), who was acquainted with the Spanish court and keen of the rare plants that thrived in the Royal Botanical Garden in Madrid^63^. She spread textual and visual information about the sunflower, and possibly also its seeds, among her network of naturalists and collectors, including the Flemish botanists Dodoens and Clusius^63^. After Dodoens^64^^: 295^ depicted the first European sunflower in his herbal in 1568, images and descriptions of this species began to circulate in manuscripts of other naturalists and physicians in Europe (e.g., Matthioli^65^^: preface^, Fragoso^66^^: title page^, Monardes^67^^: 109^ and Clusius^68^^: 14–15^). Thus, by the seventeenth century, Dutch scholars and collectors of exotic naturalia were familiar with sunflowers, which possibly promoted its cultivation at Johan Maurits’ gardens with ornamental purposes.

Interestingly, the sunflower is referenced as *Camará-guaçú,* an indigenous term from the macrolinguistic Tupi family. *Camará*, *Kamará* or *Cambará* is a generic name given to several unrelated species, such as *Lantana camara* L. (Verbenaceae) and *Ageratum conyzoides* L. (Asteraceae) (http://www.dataplamt.org.br/, accessed 2 June 2021), both found in the *Theatrum* (p. 341 and 343 respectively). According to Tibiriçá^69^, in Tupi *caa* means plant and *mbaraá* means illness, and according to Cherini^70^ *Cambará* means “leaf of rough bark”. Hence, *Camara* also refers to medicinal plants with rough leaves in general. *Guaçú* means big and *miri* small^71^, which matches with the larger inflorescence of *H. annuus* in contrast to the African weed *Sida rhombifolia* L. (Malvaceae), documented as *Camara-miri* in the HNB and “used by black people as a broom to sweep the houses of their masters”^7^^: 110^. According to the Tupi-based nomenclature associated to *H. annuus* in the *Theatrum*, Tupi indigenous groups were already familiar with the sunflower in Brazil around the 1640 s.

### Life forms and domestication status of the *Libri Picturati* plants

**Figure 4:**
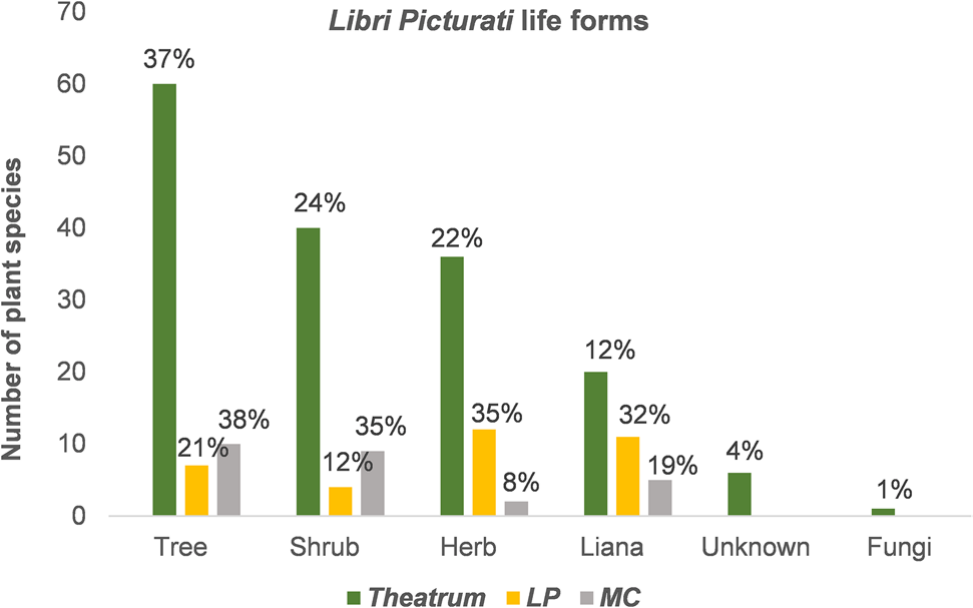
Proportion of life forms of the species depicted in the Brazilian collection of the *Libri Picturati*: *Theatrum Rerum Naturalium* (*Theatrum*), *Libri Principis* (*LP*) and *Miscellanea Cleyeri* (*MC*).

Most of the species in the *Theatrum* are tropical trees, followed by shrubs, herbs, and lianas (Fig. 4). Several are rainforest trees, such as *Andira fraxinifolia* Benth., *Garcinia brasiliensis* Mart. and *Syagrus coronata* (Mart.) Becc. The same trend was observed for the illustrations in the *MC*, with trees as the most often represented life forms, followed by shrubs, lianas and herbs. Typically, the *LP* contains much less trees, but more small herbs, shrubs and vines that were probably found in and around Mauritsstad (i.e., the former capital of Dutch Brazil, currently a part of the Brazilian city of Recife), such as *Commelina erecta* L. and *Turnera subulata* Sm., which commonly grow in disturbed landscapes.

Although the majority of the species depicted in the *Theatrum* and the *MC* are wild forest trees, some species are found both in the wild and cultivated, such as *Psidium guineense* Sw., which was part of the pre-Columbian anthropogenic forests or ‘indigenous landscape’ in Brazil^37,38,72^. Some trees were planted in or around Recife. *Hancornia speciosa* Gomes, known by its Tupi-based name *Mangabiba* or *Mangaiba* [*Mangabeira*]^7^^: 121^, was cultivated in Mauritsstad^6^^: 242,^^40^. The fruit of *H. speciosa* (*Mangaba*) was harvested in great amounts as it was a highly appreciated food^7^^: 122^. Seeds were collected to plant the tree, and Marcgrave gave details about the specific locations of varieties in different northeastern locations (Salvador, Sergipe and Olinda). *H. speciosa* was already selected and managed by indigenous groups before colonization^37^, yet wild populations of this tree are still found in the Brazilian rainforest and savannah (http://floradobrasil.jbrj.gov.br/reflora/floradobrasil/FB15558, accessed 4 June 2021).

**Figure 5:**
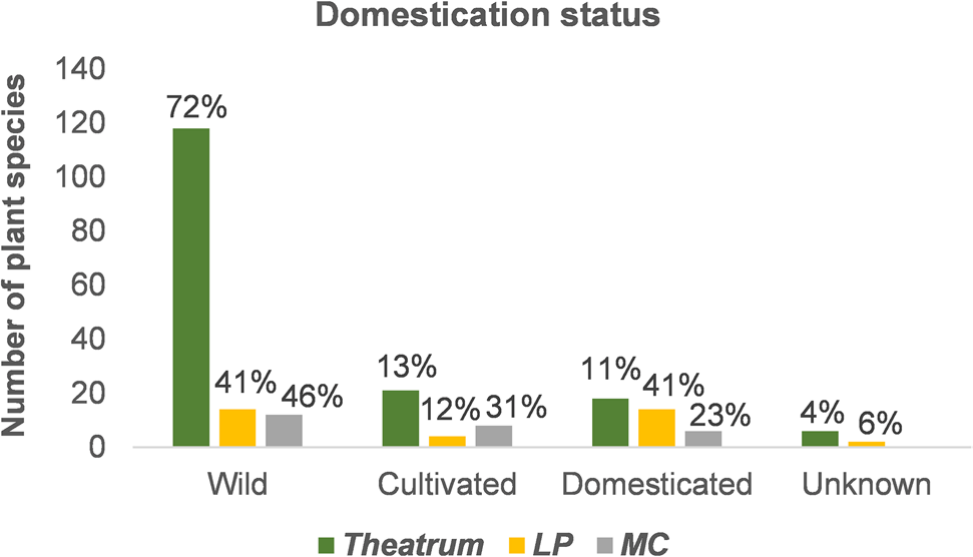
Domestication status of the species in the Brazilian collection of the *Libri Picturati*: *Theatrum Rerum Naturalium* (*Theatrum*), *Libri Principis* (*LP*) and *Miscellanea Cleyeri* (*MC*).

Domesticated plants are represented in higher proportions within the *LP* and the *MC* (Fig. 5), accounting mostly for introduced fruit species (Supplementary Dataset S1), such as *Citrus* spp., *Musa* x *paradisiaca* and *Cocos nucifera* L., which were cultivated in Maurits’ gardens in Recife^40^. The influence of the European colonization of Brazil is also visible by the presence of weeds from Asia and Africa among the illustrations in the *Theatrum* and the *LP*, such as *Abrus precatorius* L., *Argemone mexicana* L., *Boerhavia coccinea* Mill. and *Plumbago zeylanica* L. Some of these plants were introduced from Africa via the slave ships, while others may have dispersed naturally^44^. *Guilandina bonduc* L., an African scrambling shrub depicted as *Inimboi* in the *Theatrum,* was described by Piso^7^^: 95^ as “growing in abundance in sandy and dry forests of the coasts”. We categorized *G. bonduc* as a wild plant: its round seeds could have been brought by oceanic currents from West African shores and germinated in the coastal vegetation of Pernambuco and other South American areas^73^. However, *G. bonduc* may also have reached Brazil during the Trans-Atlantic slave trade, as the hard, grey seeds are used in the African game *Oware* and also used in bead ornaments^74^.

### Plant parts represented in the *Libri Picturati*

The way plants are depicted in the *Libri Picturati* provides us with information about the level of botanical skills of the artists, and how closely they worked together with the naturalists in the Dutch colony. Some plants are represented by only loose parts or depicted sterile, while others show us different organs and reproductive stages, which greatly facilitated their taxonomic identification (Table 2).

**Table 2:** Plant parts represented in the botanical illustrations of the *Libri Picturati: Theatrum Rerum Naturalium* (*Theatrum*), *Libri Principis* (*LP*) and *Miscellanea Cleyeri* (*MC*)*.*

Plant parts represented	No. of plants in the *Theatrum*	No. of plants in the *LP*	No. of plants in the *MC*
Stem^a^ + (Leaves) + Fruits + (Seeds)	73 (41%)	6 (16%)	10 (29%)
Stem + (Leaves) + Flowers + Fruits + (Seeds) + (Underground organs)^b^	38 (22%)	4 (11%)	2 (6%)
Stem + (Leaves) + Flowers	33 (19%)	16 (42%)	6 (18%)
Fruit + (Seeds) only	12 (7%)	7 (18%)	8 (23%)
Flowers only	4 (2%)	3 (8%)	3 (9%)
Sterile branches/leaves/underground organs only	15 (9%)	2 (5%)	5 (15%)

^a^Stems, including branches and trunks.

^b^Including roots, rhizomes, tubers and bulbs.

**Figure 6:**
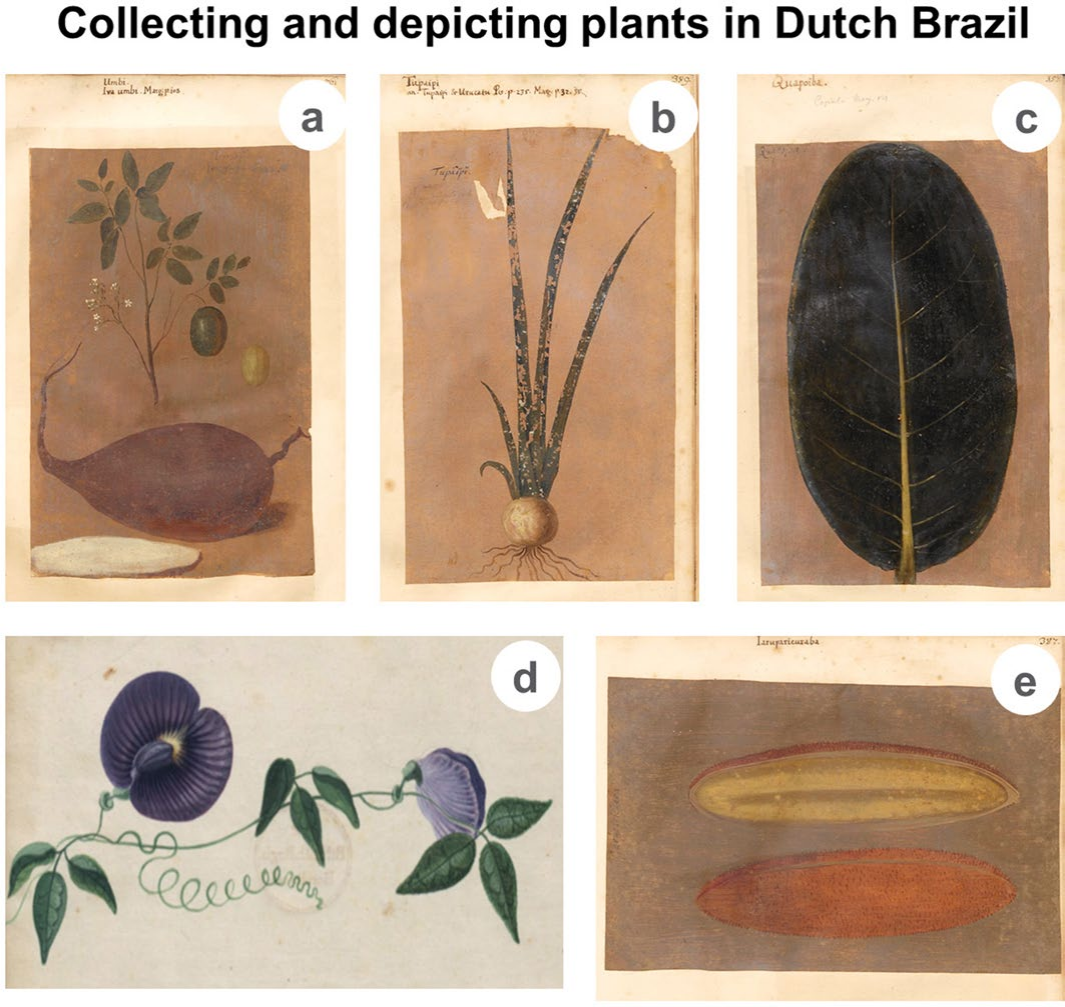
(**a**) *Spondias tuberosa* with the tuber painted in front of the branch with leaves, tiny white flowers and a detail of the immature (green) and mature (yellow) fruit in the back (*Theatrum Rerum Naturalium*: 261); (**b**) Infertile individual of *Hippeastrum psittacinum* (*Theatrum*: 389); **c** *Ficus gomelleira* leaf, probably picked from the ground (*Theatrum*: 157); (**d**) Flowering vine of *Centrosema brasilianum* (*Libri Principis*: 1); (**e**) *Amphilophium crucigerum* dry open fruit without seeds (*Theatrum*: 387).

Most illustrations depict fertile plant species with flowers and fruits, often cut in half to show the seeds, which reveals a high level of botanical knowledge. Fertile plants are more common in the *Theatrum*, in a few occasions also showing their tubers, such as *Spondias tuberosa* Arruda, known as Umbi [Iva Umbu], of which the prominent tuber in the bottom front captures the attention of the observer (Fig. 6a). Likely associated to a scientific purpose, drawing some plant parts out of proportion corresponds to a pictorial style also observed in other iconographies. This is also the case in the *Icones Plantarum Malabaricarum*, which depicts plants from Ceylon (modern Sri Lanka) in the eighteenth century and often accentuates useful fruits, flowers or roots^75^.

Piso^7^^: 78^ indicated that roots [tubers] of *S. tuberosa* deserved special attention, because of the way they developed underground and their use as a refreshment [water reservoir] for feverish patients and exhausted travelers, as he experimented himself. He and Marcgrave^7^^: 108^ also described how its fruits were valued as food. This example not only provides textual and visual evidence of the field trips to the interior by these naturalists and their first-hand experiments, but also adds insights into the connectedness between artistic and scientific practices in seventeenth century Dutch Brazil. Currently *S. tuberosa*, known as Umbu or Umbuzeiro (https://dataplamt.org.br/), is an important economic and subsistence food resource for rural communities in semiarid regions of northeast Brazil^76,77^. Its specialized root system (xylopodia) bears tubers that store liquids, sugars and other nutrients and allow the survival of the tree during the dry seasons of the *caatinga* and central Brazilian savanna, where this species is endemic^78^. The water or sweet juice of these xylopodia is still used as an emergency thirst quencher in extreme arid areas of the Brazilian *sertão*^79^; also see https://www.youtube.com/watch?v=NyGNlrljAww, accessed 25 August 2012].

In the *Theatrum*, a small proportion of plants are illustrated in their sterile stage, such as *Hippeastrum psittacinum* (Ker Gawl.) Herb. (Fig. 6b) or *Ficus gomelleira* Kunth & C.D.Bouché (Fig. 6c). Marcgrave^7^^: 32^ did not see the impressive flower of *H. psittacinum* as it is lacking in his observations^25^^: 59^. The *Theatrum* painting must have been made in the wet season in the interior of Pernambuco, when Marcgrave and the painter(s) encountered the lily with only leaves, before these fall off and make place for the mesmerizing flower^25^. *Ficus gomelleira*, depicted by a single oblong leaf with its characteristic pinnate venation (p. 157), is a large tree, up to 40 m tall (https://portal.cybertaxonomy.org/flora-guianas/node/3041, accessed 4 June 2021). It can be challenging to collect a branch, so the painter(s) or local assistants possibly picked a leaf from the ground (Fig. 6c).

The *LP* contains mainly flowering plants (e.g., *Ruellia* cf. *elegans* Poir.), tendrillate vines (e.g., *Centrosema brasilianum* (L.) Benth. (Fig. 6d)) and cultivated crops, such as peanuts (*Arachis hypogaea* L.), pumpkins (*Cucurbita pepo* L.) or guava (*Psidium guajava* L.) (Supplementary Dataset S1). Compared to the *MC* and the *LP*, a smaller proportion of the illustrations display only flowers or fruits in the *Theatrum*. Yet, these deserve special attention as the reasons for only painting the reproductive organs in the three collections may differ. While in the *MC* and *LP* flowers or fruits represent species that are domesticated or more likely to be found in urban areas, such as *Capsicum baccatum* L. or *Hancornia speciosa*, the *Theatrum* contains more loose parts of native plants found in the rainforest. *Amphilophium crucigerum* (L.) L.G.Lohmann is a liana referenced by the Tupi-related name *Iaruparicuraba* (*Theatrum*: 387) and today known in Brazil as *pente-de-macaco* (https://dataplamt.org.br/) due to its large dehiscent fruit (c.17 cm long) that opens in two valves covered with soft spines, hence its name “monkey’s comb” (Fig. 6e). Its winged seeds are not present in the drawing, possibly one empty valve was gathered from the ground, and the seeds were already dispersed by the wind.

In the *MC*, we also find some drawings of infertile structures, but these mostly belonged to species that were depicted on several folios. When assembling those folios, we observed the whole plant represented in its fertile stage: the watermelon (*Citrullus lanatus* (Thunb.) Matsum. & Nakai) is depicted with its leaves and fruit on folio 63 (verso) and its leaves on folio 64 (recto). In the case of *Furcraea foetida* (L.) Haw., whoever bounded the drawings in the *MC* collection did not realize that folios 63 (recto), 64 (verso) and double folio 68 formed together one entire plant (See Supplementary Fig. S1).

On other occasions, the painters focused on painting the plant parts that were valuable to humans. Several rainforest trees were highly valued for its edible fruits or seeds, such as *Hymenaea courbaril* L.^7^^: 101^ or *Lecythis pisonis* L., of which the “seeds (also called chestnuts) were eaten raw or roasted”^7^^: 128^ and “were considered aphrodisiacs”^7^^: 65^. The fruit of *Macoubea guianensis* Aubl. was “appreciated for its sweetness by the indigenous peoples to eat during their travels, while Europeans used it to treat chest affections”^8^^: 242^. The fruit of *Swartzia pickelii* Killip ex Ducke was “not eaten unless it was cooked, from which the inhabitants made a wholesome delicacy for the stomach called *Manipoy*”^8^^: 165^. The same applies to the tomato-like fruits of the African eggplant *Solanum aethiopicum* L., which were “eaten cooked, after seasoning with oil and pepper; it has lemon taste”^7^^: 24^. While these plants are represented in the *Theatrum* only by their fruits (Supplementary Dataset S1), the tree branches or the whole plant are depicted in the written sources. The illustrations in the books were most likely made by Marcgrave, who aimed to describe and depict as many plant parts as possible, although compromising in aesthetic aspect. The painters, on the other hand, focused on the edible parts without sacrificing their aesthetics. In any case, the illustrations from the *Theatrum* and the woodcuts and descriptions in the HNB and IURNM often complemented each other and thus facilitated our identifications.

### Current conservation status of the Brazilian species in the *Libri Picturati*

In the past centuries, the Atlantic Forest and savannah regions of northeast Brazil have been severely affected by habitat loss and degradation due to the expansion of urbanization, intensive agriculture, farming and logging^80,81^. Several plant species that were abundant enough to be noted by European artists around 1640 are not common anymore today. According to the IUCN Red List, eight species in the *Libri Picturati*, seven in the *Theatrum* and one in the *LP* are currently experiencing population decline or are at risk of facing extinction (Supplementary Table S3). Several endemic plants from the northeast Atlantic rainforest and *caatinga* biomes appear in the illustrations. Four species in the *Libri Picturati* are currently CITES-listed and restricted to trade: the cacti *Brasiliopuntia brasiliensis* (Willd.) A.Berger, *Cereus fernambucensis* Lem., *Epiphyllum phyllanthus* (L.) Haw. and *Melocactus violaceus* subsp. *margaritaceus* N.P.Taylor The latter is an endemic cactus of the coastal sand dunes’ ecoregion in the Atlantic rainforest known as *restinga*, which is severely threatened by agricultural expansion and urbanization^82^.

Some endemic species are classified as Least Concern by the IUCN or the CNC Flora (12 species), while others (13 species) have not been evaluated yet (Supplementary Dataset S1). The *MC* does not contain threatened species, but includes two endemic trees: *Attalea compta* Mart. and *Eugenia* cf. *brasiliensis* Lam., which are only found in the biodiversity hotspots of the Atlantic rainforest and the *cerrado*, both greatly affected by habitat loss^23^. The mangrove vegetation along the Brazilian coast has been severely affected by urbanization, pollution by industrial and domestic waste and climate change^83^^,^^84^, threatening the populations of the mangrove trees *Avicennia schaueriana* Stapf & Leechm. ex Moldenke and *Laguncularia racemosa* (L.) C.F.Gaertn. The occurrence of anthropogenic impacts and the lack of available data call for the implementation of more in-depth and continuous studies on the conservation status of these vulnerable populations.

### Linking the plant illustrations to the published works and Marcgrave’s herbarium

A total of 357 different plant species is described in the HNB and IURNM (Supplementary Dataset S2). Because the *Theatrum* constitutes a larger number of illustrations, we found more taxa from the books and the herbarium represented in this source (102 out of 163, 63%). However, the largest overlap was found between the *MC* and the HNB / IURNM: 21 out of 26 taxa (81%) were also described in the books. A smaller overlap exists between the *LP* and the HNB / IURNM (18 out of 34, 53%). We counted 143 taxa at species level in Marcgrave’s herbarium (Supplementary Dataset S3) and we observed some of these preserved species in all three pictorial works, with the largest percentage of taxa in common with the *MC* (seven out of 26, 27%), probably because of its smaller number of images. Strikingly, a third of the species illustrated in the whole Brazilian collection of the *Libri Picturati* could not be ascribed to the species described by Marcgrave or Piso (61 out of 180, 34%).

## Discussion

Mentzel intended to include much more botanical illustrations in the *Theatrum*, as is shown by the empty folios with the Tupi vernaculars and the references to plants described by Marcgrave and Piso. The combination of the plant illustrations in the *Libri Picturati* and the written sources provide a more complete overview of the flora as perceived by naturalists and painters in the Dutch colony than the published works alone. The links provided between the published and unpublished images allow for more in-depth studies by (art) historians, botanists and other scholars interested in the floristic landscape of Dutch Brazil.

As highlighted by Whitehead and Boeseman^3^, the study of these plant images allows us to trace the introduction of exotic species by humans; whether they were Portuguese or Dutch colonists growing popular fruits or ornamentals, Jesuits bringing their plant-based remedies from Europe or enslaved Africans planting crops from their homeland. This shows the diverse context of Dutch Brazil, where Portuguese, Dutch, French, Sephardic Jews, and diverse indigenous and African ethnicities were cohabiting at the time when Marcgrave and Piso collected their information. Considering this social diversity, the iconographic identifications provide evidence for the first records of some introduced species in Brazil, such as *Xylopia aethiopica* in the *Libri Picturati*, which reveals the presence of this plant for the first time in the Neotropics*.* This adds new insights into the corpus of literature on plant exchange related to the African diaspora in the Americas and the preservation of its botanical legacy by African descendants^43,44,85–87^. The sunflower in the *Theatrum* proves that it was introduced to Brazil at least 300 years earlier than previously thought^58^. The Tupi-related names for this exotic plant show how indigenous people incorporated the foreign flora and reveals the role the Dutch played in introducing plants to Brazil; sometimes promoted by the early plant exchange between Spain and the Netherlands.

The differences in plant habit, domestication status and plant parts represented between the *Theatrum*, *LP*, and *MC* reveal the methods of selection and collection by the group of naturalists and artists that collaborated in the three visual collections. The *MC* shares characteristics with both the *Theatrum* (many wild trees with fruiting branches) and the *LP* (multiple small weeds and separate fruits of domesticated crops). Most of these plant parts also figure in the art works that were possibly made after them, such as Eckhout’s still-life paintings and the series of tapestries of the Old Indies^3,14,87^. The plants represented in these paintings could have been found in Recife’s surroundings and close to the court painters in Mauritsstad^14,19^. The same may apply to the makers of the *LP* illustrations, who probably stayed in an open, anthropogenic vegetation, as evidenced by the many herbs and weeds depicted. Contrary to previous expectations, the large number of rainforest trees in the *Theatrum* show that the artist(s) either have accompanied Marcgrave during his botanical expeditions, went into the hinterland themselves or obtained the plant material from locals.

Many of the *Theatrum* illustrations do not only represent species described and / or depicted in the HNB and IURNM, but they resemble, sometimes in an almost identical way, the woodcuts in Marcgrave and Piso’s published sources (forthcoming paper). The evidence of the *Theatrum* illustrations as sources for the engravings have been discussed by Whitehead and Boeseman^3^ and by Brienen^14^, but the reasons for leaving out some of these botanical images in the written sources have been overlooked. Editor De Laet must have had difficulties to link some of these images to the descriptions, as parts of Marcgrave’s notes were presumably missing and his sudden and mysterious death prevented him fromdiscussing the arrangement of the HNB content with De Laet. Marcgrave was a skilled botanist, who aimed for full descriptions of plants, capturing as much as possible the morphology, life stages, phenology and other characters. It is likely that he excluded some of the specimens known only by loose parts, such as the fruits of *Amphilophium crucigerum* or the flower of *Neomarica* cf. *northiana* (Schneev.) Sprague (Supplementary Dataset S1). These loose flowers and fruits were either found by him during his expeditions, or brought to him by local people or artists, but never observed in situ by himself in their complete form. Hence, he did not include them in his descriptions, as Marcgrave himself stated: “I will not write anything down that I have not seen or observed myself”^7^^: 139^. It is not surprising that most of the *MC* plants are also found in the HNB and IURNM, as these represent plants that grew in Maurits’ palace gardens or surroundings, and also figure in Eckhout’s paintings that were aimed to represent the territory in the colony as seen by the European settlers^14^. The *LP* contains many common weeds from roadsides and gardens, likely admired by the artist who depicted them for the beauty of their flowers or fruits, which fulfilled its contemplative purposes^16^, but probably dismissed by scientists like Marcgrave and Piso. In our forthcoming paper, we will use the correlations between the *Libri Picturati* and Marcgrave and Piso’s books and herbarium to shed light into the origin of the woodcuts and the multiple connections between visual and textual sources as scientific and artistic entities in the early modern period.

The joint study of all associated materials has facilitated our identifications of the flora represented in both visual and textual sources. In the *MC*, *Citrus* x *aurantiifolia* (Christm.) Swingle*,* of Asian origin, was first mistaken for a native Brazilian fruit due to the lack of coloration and other key characters. We found a plant drawing in Wagener^27^^: 5^ that bears great resemblance to the sketch in the *MC*, but it is colored dark green and includes a brief description and the vernacular name *Sweet lemon*, which dissipated any doubts about this taxon. Attempts to reveal the species behind the three separate folios that together depict *Furcraea foetida* were made before, but the pieces were not assembled properly, or the plant was mistakenly considered a Bromeliad^20^*.* Already known by the Spaniards who encountered this plant in their colonies in the Caribbean, *F. foetida* was thought to be brought in 1648 from Spain to the Netherlands^88^. However, as the *MC* painting reveals, the Dutch already knew this plant around 1640 and cultivated it in northeast Brazil to obtain its fibers^31^. *Furcraea* species have been used as fiber sources by multiple indigenous groups; a traditional use appropriated by Europeans and now in disuse in the western world^89^. However, the ecological footprint of its introduction is still present, as *F. foetida* is today an invasive species along the Atlantic Brazilian coast^90^. These new botanical findings not only provide new insights on vegetation distribution and dispersion; they reveal baseline-conditions where the environment had started being altered by intensive western agriculture and other high profit enterprises of early capitalist societies. These plant illustrations act as a repository of historical distributional data that allows us to trace the origins of ecological disturbance^91^, in this case, in northeastern Brazil since the seventeenth century. Moreover, with these findings we add valuable information to the existent corpus of literature on these taxa (e.g., for *Furcraea* spp.^31,90,92^).

One of the most relevant new fields in ethnobotany is plants & art research^93^. The digitalization of the Brazilian collection of the *Libri Picturati* enabled us to take this innovative approach and perform an in-depth botanical study of its beautiful plant images. Through this study, we draw attention to the relevance of digitizing and studying historical collections, as well as facilitating its access to a larger and more diverse community^94^. As proven throughout this paper, these valuable but sometimes forgotten collections are great sources of data for cultural and biodiversity research^95^.

In summary, through the access to these digital natural history collections and their scientific study, we disclosed a valuable source of data that can be researched from several perspectives. By identifying the depicted plants, we revealed the various habits, geographical origin and domestication status. By analyzing the plant parts depicted and comparing all visual and textual sources, we revealed the methods of collection and collaboration between botanists and artists in Dutch Brazil. We detected plants that are no longer abundant in northeast Brazil due to deforestation, urbanization and large-scale agriculture, and drew attention to their urgent conservation needs. We hope that the *Libri Picturati*—as a botanical and cultural treasure—will reach the inhabitants of the country where these illustrations were made, and that the Brazilian vegetation will continue to reflect the beauty and rich diversity that was captured by artists and naturalists almost 380 years ago.

## Supplementary Information

Supplementary Information 1.

Supplementary Information 2.

Supplementary Information 3.

Supplementary Information 4.

Supplementary Information 5.

Supplementary Information 6.

Supplementary Information 7.

## Data Availability

Most of the data generated or analyzed during this study are included in this published article (and its Supplementary Information files and links: see methods). The botanical illustrations of the *Theatrum Rerum Naturalium* are available from the Jagiellonian library in Krakow but restrictions apply to the availability of these data, which were used under license for the current study, and so are not publicly available. Data are however available from the authors upon reasonable request and with permission of the curators of the Jagiellonian library in Krakow.
