# Supplementary figures and images for: Looking into the flora of Dutch Brazil: botanical identifications of seventeenth century plant illustrations in the Libri Picturati
Source: Sci Rep. 2021 Oct 5;11:19736. doi: 10.1038/s41598-021-99226-8 (PMC8492696; doi:10.1038/s41598-021-99226-8)

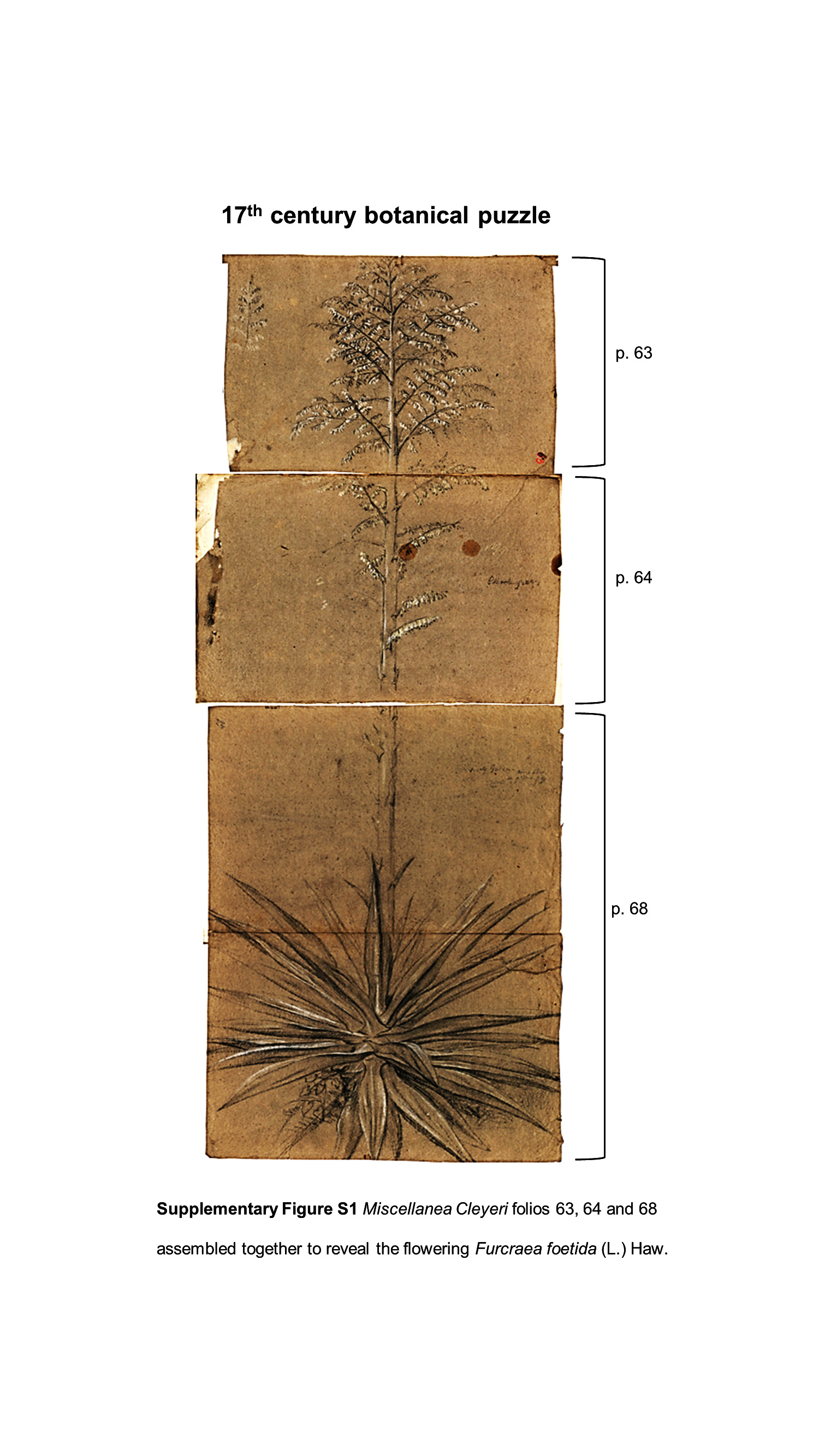

Supplement: Supplementary file 4 — Supplementary Information 4. [file 41598_2021_99226_MOESM4_ESM.jpg]
