# Supplementary material for: Looking into the flora of Dutch Brazil: botanical identifications of seventeenth century plant illustrations in the Libri Picturati
Source: Sci Rep. 2021 Oct 5;11:19736. doi: 10.1038/s41598-021-99226-8 (PMC8492696; doi:10.1038/s41598-021-99226-8)
Supplement: Supplementary file 5 — Supplementary Information 5. [file 41598_2021_99226_MOESM5_ESM.pdf]

Supplementary Table S1 Content and plant taxa identified in the *Libri Picturati's* Brazilian plant collection.

Theatrum Rerum Naturalium
735 folios numbered (1-735), verso always blank
1-731 folios with illustrations, vernacular names, references and blank folios
733-735 folios with Index *Plantarum Brasiliae*
366 folios (recto) plus one glued folio between 729 and 731
15 folios completely blank
One folio with only a vernacular name (Ambaibuna) (Fig. 1)
190 folios without illustrations but with 220 vernacular names and references
30 folios with two vernacular names (from two taxa) per folio
205 taxa with 18 vernacular names that occur twice
197 taxa identified to species level, five to genus level and three unidentified
160 folios with Brazilian plant illustrations
172 Brazilian plant illustrations that correspond to 176 plant images
12 folios with two figures per folio, one folio with three taxa (f. 37), two folios with two taxa (f. 341, 541)
11 taxa depicted twice and one taxon depicted three times
163 taxa identified: 150 identified to species level, eight to genus level, five unidentified
Libri Principis
34 folios with plant illustrations that correspond to 38 plant images
Four folios with two taxa per folio
35 plant names: 17 Latin names (13 unique) and 18 vernacular names (16 unique)
32 plant images identified to species level and six plants identified to genus level
Four species depicted twice

34 taxa identified: 29 taxa identified to species level and five taxa identified to genus level
Miscellanea Cleyeri
28 folios with plant illustrations that correspond to 34 plant images<br>One folio with two figures per folio (f. 55), four folios with one taxon<br>Two folios with two taxa depicted, one folio with three taxa depicted (f. 57)<br>35 plant names: 17 Latin names (13 unique), 18 vernacular names (16 unique)<br>32 plants identified to species level and two plants identified to genus level<br>Three species depicted twice, one species depicted three times, one species depicted four times<br>26 taxa identified: 24 identified to species level and two taxa identified to genus level
