# Supplementary material for: Looking into the flora of Dutch Brazil: botanical identifications of seventeenth century plant illustrations in the Libri Picturati
Source: Sci Rep. 2021 Oct 5;11:19736. doi: 10.1038/s41598-021-99226-8 (PMC8492696; doi:10.1038/s41598-021-99226-8)
Supplement: Supplementary file 6 — Supplementary Information 6. [file 41598_2021_99226_MOESM6_ESM.pdf]

Supplementary Table S2 Origin of the introduced species that were present in Dutch Brazil c. 1640 and depicted in the *Libri Picturati*.

Origin	Plant Species
**Tropical Asia - Pacific**	*Abrus precatorius* L.
	Citrus x aurantiifolia (Christm.) Swingle
	Citrus x aurantium L.
	Citrus x *limon* (L.) Osbeck
	Cocos nucifera L.
	Dioscorea cf. *alata* L.
	Musa × *paradisiaca* L.
	Plumbago zeylanica L.
	Cf. *Plumeria* sp.
**Tropical Africa**	*Abelmoschus moschatus* Medik.
	Citrullus lanatus (Thunb.) Matsum. & Nakai
	Guilandina bonduc L.
	Lagenaria siceraria (Molina) Standl.
	Ricinus communis L.
	Solanum aethiopicum L.
	Tamarindus indica L.
	Xylopia aethiopica (Dunal) A.Rich.
**South Africa**	*Zantedeschia aethiopica* (L.) Spreng.
**Middle East, South Africa**	*Aloe vera* (L.) Burm.f.
**S-Europe, Middle East (Mediterranean basin)**	*Punica granatum* L.
	Vitis vinifera L.

**US, Mexico**	*Cucurbita pepo* L.
	Helianthus annus L.
**US, N-South America**	*Boerhavia coccinea* Mill.
**Peru (Andes)**	*Gossypium barbadense* L.
**Central America, Caribbean**	*Argemone mexicana* L.
	Carica papaya L.
	Ipomoea quamoclit L.
	Psidium guajava L.
	Tagetes cf. *erecta* L.
	Zea mays L.
**Central America, Andes-N- South America**	*Furcraea foetida* (L.) Haw.
	Ipomoea batatas (L.) Lam.
	Phaseolus vulgaris L.
	Phaseolus sp.
