# Supplementary material for: Looking into the flora of Dutch Brazil: botanical identifications of seventeenth century plant illustrations in the Libri Picturati
Source: Sci Rep. 2021 Oct 5;11:19736. doi: 10.1038/s41598-021-99226-8 (PMC8492696; doi:10.1038/s41598-021-99226-8)
Supplement: Supplementary file 7 — Supplementary Information 7. [file 41598_2021_99226_MOESM7_ESM.pdf]

Supplementary Table S3 Conservation status of plant species from the *Libri Picturati* currently categorized as threatened by anthropogenic disturbance.

	Conservation status	***Endemic Region***	***Threats[a]***
Aechmea muricata	Endangered	Atlantic Rainforest	Urbanization
Hippeastrum psittacinum	Endangered	Atlantic Rainforest	Mining, harvesting (ornamental, medicinal)
Pilocarpus jaborandi	Endangered	Atlantic Rainforest, Caatinga	Fragmented population, deforestation, harvesting (medicine)
Melocactus violaceus subsp. margaritaceus	Vulnerable: Decreasing / CITES-listed	Atlantic Rainforest	Urbanization, road construction, agro-industry
Bowdichia virgilioides	Near Threatened		Logging, ornamental trade
Avicennia schaueriana	Least Concern: Decreasing		Urbanization, pollution, agri- /aquaculture, logging
Brasiliopuntia brasiliensis	Least Concern: Decreasing / CITES-listed		Urbanization, agriculture, mining, logging
Laguncularia racemosa	Least Concern: Decreasing		Urbanization, industries, agriculture, aquaculture, logging, pollution

[a] Threats retrieved from http://cncflora.jbrj.gov.br/ and https://www.iucnredlist.org/.
